# Exploring the Dynamic Nexus Among Economic Growth, Industrialization, Medical Technology, and Healthcare Expenditure: A PMG-ARDL Panel Data Analysis on Income-Level Classification Along West African Economies

**DOI:** 10.3389/fpubh.2022.903399

**Published:** 2022-06-17

**Authors:** Evelyn Agba Tackie, Hao Chen, Isaac Ahakwa, Samuel Atingabili

**Affiliations:** School of Management, Jiangsu University, Zhenjiang, China

**Keywords:** healthcare expenditure, economic growth, medical technology, industrialization, West Africa

## Abstract

This article explored the dynamic nexus among economic growth, industrialization, medical technology, and healthcare expenditure in West Africa while using urbanization and aged population as control variables. West African countries were sub-sectioned into low-income (LI) and lower-middle-income (LMI) countries. Panel data extracted from the World Development Indicators (WDI) from 2000 to 2019 were used for the study. More modern econometric techniques that are vigorous to cross-sectional dependence and slope heterogeneity were employed in the analytical process in order to provide accurate and trustworthy results. The homogeneity test and cross-sectional dependency test confirmed the studied panels to be heterogeneous and cross-sectionally dependent, respectively. Moreover, the CADF and CIPS unit root tests showed that the variables were not integrated in the same order. This, thus, leads to the employment of the PMG-ARDL estimation approach, which unveiled economic growth and urbanization as trivial determinants of healthcare expenditure in the LI and LMI panels. However, the results affirmed industrialization as a major determinant of healthcare expenditure in the LI and LMI panels. Additionally, medical technology was confirmed to decrease healthcare expenditure in the LMI panel, whereas in the LI panel, an insignificant impact was witnessed. Also, the aged population was found to intensify healthcare expenditure in both the LI and LMI panels. Lastly, on the causal connection between the series, the outcome revealed a mixture of causal paths among the variables in all the panels. Policy recommendations have therefore been proposed based on the study's findings.

## Introduction

Healthcare is a necessity for all humanity. In recent years, life expectancy has increased as individuals live longer than ever before because of improved living conditions, advanced medical technology, and other factors (1). Anticipations of healthcare have risen, as the sector has developed and advanced technologically. As a result of the current type of jobs and lifestyle choices, many people have become physically inactive, and healthcare needs are intensifying (2). As people's healthcare needs grow, healthcare spending is also expected to increase to meet those needs (3). In most developed economies, healthcare expenditure rises faster than income growth, i.e., the healthcare expenditure in the European Union, OECD countries, and G7 countries has been increasing steadily over the years (4). Healthcare expenditure is defined as spending on health-related goods and services and financing medical facilities (5). Healthcare spending has increased dramatically worldwide in recent years, with overall spending rising from US$ 3.5 trillion in 1995 to US$ 8 trillion in 2016 (6). This equates to about 4% yearly growth over the last 20 years. The Institute for Health Metrics and Evaluation (IHME) asserts that healthcare expenditure might increase globally from $ 8 trillion in 2018 to $ 18 trillion in 2040, with healthcare accounting for about 9% of GDP globally by 2040. Adding resources to assist the health system is one of the main issues among various stakeholders in the midst of the COVID-19 pandemic. Exploring the role of healthcare expenditure is an important topic in health-economics studies.

Since 2000, healthcare expenditure in the West African region has been increasing dramatically as a result of numerous health concerns that countries in this region are facing (7). The Human Development Index classifies most nations in West Africa as having a low-medium life expectancy, exacerbated by the region's high illness burden, particularly malaria, which costs the region's economy about 132 billion dollars each year (8). Also, over the past decades, there has been a significant rise in the disease burden of non-communicable diseases in West Africa, which has been attributed to an increase in the prevalence of cardiovascular risk factors such as unhealthy diets, inadequate physical exercise, high blood pressure, weight gain, diabetes, hyperlipidemia, and air quality. Non-communicable illnesses account for 37% of all economic losses in West Africa, according to the World Health Organization (9). In comparison, 27% of the economic losses stem from communicable diseases such as tuberculosis (TB) and AIDS (9). By 2030, non-communicable diseases will surpass communicable, maternal, neonatal, and nutritional diseases as the primary cause of death in West Africa (10). These causes of increased health challenges in the West African region are driven by industrialization, urbanization, population growth, aged population, and increased living standards (11). The prevalence of non-communicable diseases has raised both government and individual out-of-pocket healthcare spending in West African economies (10). Using the most current available cross-sectional data, this article empirically examines some of the drivers of healthcare expenditure in West African nations. While several econometric studies have been conducted on healthcare expenditure in OECD nations, the United States, and other developed countries, the literature has a striking dearth of research on healthcare expenditure in West African countries, thus making West African economies an excellent sample for empirical research.

Several academicians have researched the roles of economic activities in healthcare expenditure. Economic growth and healthcare spending are interdependent according to Raghupathi and Raghupathi (12). Zaidi and Saidi (13) assert that economic progress increases healthcare spending. Baioumy et al. (14) suggest that boosting a country's economic growth has the ability to increase its domestic healthcare spending, since accelerated economic growth is preceded by the country's economic progress, thereby improving its national health issues. Researchers like Haseeb et al. (15) discovered a substantial and positive influence of economic progress on healthcare spending on the long term effect, while on the short-term effect, there was an insignificant impact of economic growth on healthcare spending specifically for ASEAN economies. Nevertheless, Chireshe and Ocran (16) conducted research on sub-Saharan African countries and found that government healthcare spending and economic growth are negatively connected. Again, Jakovljevic et al. (17) maintain that economic growth has a negative but statistically significant consequence on healthcare spending in Emerging Markets Seven (EM7) countries. The conflicting outcomes on economic growth and healthcare spending necessitate further investigations on the two variables.

Industrialization is another determinant of healthcare spending. Abban and Hongxing (18) assert that industrialization is the process of changing an agrarian economy into a manufacturing one. This transformation results in harmful substances like carbon dioxide being emitted into the atmosphere (19). Emissions produced by manufacturing firms enter the human body through the lungs or skin and cause all forms of diseases (20). Raheem and Ogebe (21) stated that industrialization brings about change in the way people think and conceptualize problems associated with the environment. Therefore, selecting industrialization as a determinant of healthcare spending is essential to this research.

Zhou et al. (5) analyzed the impacts of medical technology on health spending using growing economy panel data from 2000 to 2018. The empirical findings show that medical technology, measured by patent applications, impacts rising healthcare expenditure. Shakoor et al. (22) found that one variable that fosters healthcare spending in the Pakistani economy is medical technology, measured by the life expectancy rate at birth. Using time-series data from 1960 to 2012, Murthy and Okunade (23) investigated factors contributing to healthcare spending. The researchers used the ARDL method to derive their results. It was discovered that the most influential factor that contributes to healthcare spending in the United States is medical technology. Nonetheless, more research has to be performed on how medical technology can influence healthcare spending.

In 1950, 30% of the world's populace resided in urban areas. In 2015, the figure reached 54% globally, and by 2030, the percentage is projected to increase to 60 according to the World Health Organization. Countries with low and medium incomes bear the largest percentage of people living in urban areas according to Çetin and Bakirtaş (24). The urban population's annual change rate was estimated at around 2.16% between 1995 and 2015 according to the 2016 United Nations Human Settlement Program report. Around the same time, the average yearly rate variation was estimated at 0.88, 2.63, and 3.68% for high-income, middle-income, and low-income countries, respectively. Without a doubt, the fast-growing population has substantial economic, health, and environmental implications, particularly for developing countries compared to advanced countries. Third-world countries are confronted with health-related issues as urbanization increases, which has become one of the major challenges of socioeconomic development. Therefore, selecting urbanization as a control variable is critical in the West African region.

To date, the effect the aged population has on healthcare spending is still not known. Shakoor et al. (22) assert that globally, the aged population is likely to increase by 56%; thus, there could be a steady growth from 901 million to 1.4 billion. These numbers can surpass the number of children between the ages of 0 and 9. Healthcare costs for the elderly are quite nearly two times that of other age categories (25). According to Ha et al. (26), a more than 20% increase in healthcare spending for the overall cardiovascular disease is recorded among the aged population. Furthermore, Lopreite and Zhu (27) discovered that an aged population has a sturdy effect on healthcare spending. According to Borrescio-Higa and Valenzuela (28), countries with low literacy levels are more likely to face a consistent increase in healthcare spending than countries with higher literacy rates. Other researchers like Costa-Font and Vilaplana-Prieto (29) affirmed that healthcare spending is not dependent on the aged population. Baharin and Saad (30) also found similar results that aged population does not escalate healthcare expenditure. Notwithstanding, these conflicting outcomes between aged population and healthcare expenditure require further investigation of the two variables. Therefore, the selection of aged population as a control variable is vital to this research.

This current research explores the predictors of healthcare spending relying on the above literature. This research adds to the current body of knowledge by analyzing the dynamic impact of economic growth, industrialization, and medical technology on healthcare expenditure while considering aged population and urbanization as control variables across West African countries. As a result, in three ways, the research sought to level the gaps in prior research findings and was aimed at West Africa with the full panel and sub-panel procedures: First, the study adds to the extant research on healthcare spending and its predictors. Our research is one of the first to look at factors that influence healthcare spending in West Africa. Second, the econometric techniques used in this study differ significantly from those used in previous studies. Although there are a handful of studies on healthcare spending and other indicators, hardly any of them looked into issues concerning heterogeneity and residual cross-sectional correlations. Quite often, such studies assume homogeneity or independent residual correlations. Founded on cross-sectional independence and homogeneity assumptions, if panel data are heterogeneous and cross-sectionally dependent, it will probably lead to inaccurate and misleading prediction results. Therefore, we investigated whether the panel data utilized in this current research were cross-sectionally dependent and heterogeneous. Finally, most studies on healthcare spending and other indicators are conducted from a regional or panel perspective and with little consideration for disparities across sub-regional groups. Therefore, this study addresses the situation by classifying the sampled panel countries in West Africa into two separate subgroups: those with low-income and lower-middle-income levels according to the World Bank categorization. The sub-categories are intended to explore if features unique to the above said income categorizations affect healthcare spending and its recommended drivers in West African countries.

The remaining sections of the study are subsequently organized as specified: The Methods and material section presents the methodological approach used in this current study. The empirical results summary is found in the subsequent section. The last section contains the discussion, policy implications, limitations, and recommendations of the study.

## Methods and Materials

### Data Source and Descriptive Statistics

A panel of 16 West African countries was involved in this research and was divided into lower-middle-income (LMI) and low-income (LI) countries according to the World Bank's classification of countries. Table 1 illustrates the countries' classifications in the LI and LMI panels. To make up for shortcomings in previous research, the effects of independent factors on healthcare expenditure were robustly assessed in these sub-categories and the whole population using STATA (version 15.0). The research used data from the World Development Indicators (WDI). Table 2 contains additional information on the series used for the study.

**Table 1:** Classifications of countries in West Africa.

**Panel**	**Countries**
Low income	Burkina Faso, Gambia, Guinea Bissau, Liberia, Guinea, Sierra Leone, Niger, Mali, and Togo.
Lower-middle income	Ghana, Ivory Coast, Senegal, Mauritania, Cape Verde, Nigeria, and Benin,
Whole sample	Ghana, Ivory Coast, Senegal, Burkina Faso, Sierra Leone, Guinea, Gambia, Liberia, Mali, Mauritania, Guinea Bissau, Niger, Cape Verde, Nigeria, Benin, and Togo.

**Table 2:** Data source and variable definition.

**Variables**	**Abbreviations**	**Definitions**	**Source**	**Period**
Total healthcare expenditure	HCE	Current US $	WDI	2000–2019
Economic growth	GDP	GDP per capita (constant 2010 US$)	WDI	2000–2019
Industrialization	IND	Industry (including construction), value added (constant 2010 US$)	WDI	2000–2019
Medical technology	MT	Life expectancy	WDI	2000–2019
Urbanization	URB	Percentage of total population	WDI	2000–2019
Aged population	AGP	Individuals above 65 years old	WDI	2000–2019

### Model Formulation

This study used HCE as a proxy for healthcare expenditure, which is the response variable. The vector of explanatory variables, on the other hand, included economic growth (GDP), aged population (AGP), medical technology (MT), urbanization (URB), and industrialization (IND). The following econometric equation was assessed for the above variables and their proxies:


(1) $$\begin{array}{r} \;HCE=\alpha_{i}+\beta_{1}GDP_{it}+\\ \beta_{2}IND_{it}+\beta_{3}MT_{it}+\beta_{4}URB_{it}+\beta_{5}AGP_{it}+\mu_{it} \end{array}$$

where *it* indicates a non-observed time-invariant specific effect; β_1_, β_2_, β_3_, β_4_ and β_5_ correspond to GDP, IND, MT, URB, and AGP effects on HCE; μ_*it*_ is the presumed error term with an average of zero and a variation of σ^2^; *i* means the numeral of nations (*i* = 1, 2, 3..., *N*); *t* represents the timeline (*t* = 1, 2, 3..., *T*); α_*i*_ indicates the constant term. To eliminate heteroscedasticity and data fluctuations, logarithms were applied to the variables. Musah et al. (31), Chen et al. (11), and Musah et al. (32) claimed that results obtained from log-linear models are more consistent than the results of simple linear models. As a result, Equation 1 was converted to a logarithm model;


(2) $$\begin{array}{r} \begin{array}{c} \begin{array}{l} \;lnHCE=\alpha_{i}+\varphi_{1}lnGDP_{it}+\\ \varphi_{2}lnIND_{it}+\varphi_{3}lnMT_{it}+\varphi_{4}lnURB_{it}+\varphi_{5}lnAGP_{it}+\mu_{it} \end{array} \end{array} \end{array}$$

where α_*i*_, μ_*it*_, *i*, and *t* are already outlined in Equation 1; lnGDP, lnAGP, lnMT, lnURB, lnIND, and lnHCE are GDP, IND, MT, URB, AGP, and HCE log transformations, respectively; and φ_1_, φ_2_, φ_3_, φ_4_ and φ_5_ are the lnHCE elastics concerning lnGDP, lnIND, lnMT, lnURB and lnAGP transformations, respectively. For the study, φ_1_ and φ_2_ were predicted to influence HCE positively, while φ_4_ was expected to have negative impacts on HCE. However, φ_3_ and φ_5_ might have an adverse or positive effect on HCE.

### Econometric Approaches

#### Cross-Sectional Dependence and Slope Heterogeneity

As trade and other economic activities tie West African countries, it is necessary to determine whether the series employed was cross-sectional-dependent or not. Ignorance of cross-sectional dependence may lead to spurious and misleading findings (33–35). As a result, this study conducted Pesaran's (36) cross-sectional reliance test, Pesaran's (37) cross-sectional dependency test, the Lagrangian multiplier test by Breusch and Pagan (38), and Friedman's (39) cross-sectional dependency test to determine whether or not cross-sectional reliability existed in the panel. The null hypothesis of cross-sectional independence was evaluated with the alternative hypothesis of cross-sectional dependence. The rejection of the null hypothesis indicated the presence of cross-sectional dependence among the selected variables.

Second, to check whether or not heterogeneity exists, we used the homogeneity test developed by Pesaran and Yamagata (40). This was because ignoring slope heterogeneity might prejudice the regression analysis and lead to wrong tests of hypotheses (41–43). The null hypothesis of homogeneity was evaluated with the alternative hypothesis of heterogeneity. The rejection of the null hypothesis indicated slope heterogeneity among the selected variables. According to Ntarmah et al. (44) and Chen et al. (11), assessing for residual cross-sectional correlations and homogeneity is important for the choice of additional econometric assessments.

#### Unit Root

It is hypothetically presumed that a unit root process will define study variables because the period of the panel data utilized is rather long (45–47). Relying on the presumption mentioned above and the possibility of cross-sectional dependence, appropriate panel unit root tests such as Pesaran's (48) cross-sectionally augmented IPS (CIPS) and cross-sectionally augmented Dickey-Fuller (CADF) were performed to determine the stationarity of the series involved in the study. Remarkably, the regression analysis test is performed to examine the characteristics of the series when the unit root tests reveal that the data series lacks a unit root. As a result, a co-integration test is performed if variables show integration in the same order. Based on the assumptions mentioned above, this study demonstrated that its panel data lacked longevity, which can detect the unit root outcome because not all series were combined in a similar order (mix integration order), suggesting that a co-integration relationship was not detected.

#### ADRL Model

The panel ARDL model promulgated by Pesaran et al. (49) was employed in this research to assess the long-run association among the variables healthcare expenditure, economic growth, aged population, industrialization, urbanization, and medical technology. The use of the approach mentioned above is backed by Pesaran et al. (1999), who stated that the ARDL is suitable in a case where the series integration order is single or mixed. This technique also aids in consistent and accurate estimation by removing the issue of endogeneity.

#### Causality

Finally, the path of causal associations amid the study variables was performed by Dumitrescu and Hurlin (50) heterogeneous panel Granger causality test. When conducting a Dumitrescu and Hurlin (50) panel causality test, the null hypothesis is homogeneously non-causality, which asserts that no causal relationship exists between the sequences in any cross-section of the panel's data set. This test was chosen because it provides consistent and trustworthy findings when cross-sectional dependency and slope heterogeneity are present. The Dumitrescu and Hurlin (50) test permits coefficients to be varied across cross-sections in the following manner:


(3) $$\begin{array}{r} a_{0},\;i\neq a_{0},\;j,a_{1},i\neq a_{1},\;j,\ldots ,\;a_{n},\;i\neq a_{n},\;j,\forall i,j \end{array}$$

(4) $$\begin{array}{r} b_{1},\;i\neq b_{1},j,\;\ldots ,\;b_{n},\;i\neq b_{n},\;j\forall i.j \end{array}$$

The test was then conducted using the simplest version of the Granger causality analysis for each cross-section. Following that, the test data were averaged. Wbar is the abbreviation for this test statistic, while the standardized form is the abbreviated Zbar.

### Empirical Result and Analysis

#### Preliminary Result

Table 3 shows descriptive statistics of the research factors. In the LI, LMI, and whole sample panels, industrialization had the greatest mean (M) figure of 20.066, 19.246, and 19.708, respectively, with a corresponding standard deviation (SD) of 1.317, 6.526, and 4.437, respectively, followed by aged population with (M = 12.078, SD = 0.856), (M = 12.746, SD = 1.499), and (M = 12.372, SD = 1.224), respectively; medical technology (M = 4.017, SD = 0.087), (M = 4.088, SD = 0.115), and (M = 4.047, SD = 0.106), respectively. Also, in the whole sample, in the LMI and LI panels, urbanization had (M = 3.679, SD = 0.322), (M = 3.855, SD = 0.141) and (M = 3.545, SD = 0.353), respectively, and economic growth had (M = 3.554, SD = 0.242), (M = 3.57, SD = 0.099), and (M = 3.542, SD = 0.311), respectively. Lastly, in terms of the whole sample, healthcare expenditure had a mean figure of 3.669 with an SD of 0.636, and in the LMI and LI panels, healthcare expenditure recorded (*M* = 3.997, SD = 0.608) and (*M* = 3.421, SD = 0.524), respectively. The kurtosis and skewness of the variables are further shown in Table 3. The table shows the negative skewness of all the variables in the LI panel. In addition, all the study variables exhibited wider ends with positive excess kurtosis (K > 3) in the LI panel except for the aged population and industrialization, which had narrow ends with negative excess kurtosis (K <3). The distribution of aged population, medical technology, and industrialization in the LMI panel was negatively skewed, but that of healthcare expenditure, economic growth, and urbanization was positively skewed. In the LMI panel, the distribution of economic growth and industrialization exhibited larger ends with positive excess kurtosis (K > 3). However, narrower ends with negative excess kurtosis (K <3) were recorded in healthcare expenditure, aged population, urbanization, and medical technology. Lastly, in the whole sample, economic growth, urbanization, medical technology, and industrialization were negatively skewed, with values of −11.705, −1.345, −0.241, and −2.714, respectively. Conversely, healthcare expenditure and aged population were positively skewed. Again, the distribution of all the study variables had wider ends with positive excess kurtosis (K > 3) in the whole panel.

**Table 3:** Descriptive statistics and correlation analysis of the study variables.

	**Statistic**	**lnHCE**	**lnGDP**	**lnAGP**	**lnURB**	**lnMT**	**lnIND**
LI	Mean	3.421	3.542	12.078	3.545	4.017	20.066
	Std. dev	0.524	0.311	0.856	0.353	0.087	1.317
	Variance	0.275	0.097	0.733	0.124	0.008	1.735
	Min	2.042	−0.157	10.428	2.784	3.675	17.219
	Max	4.949	4.050	13.314	4.126	4.161	22.132
	Skewness	−0.107	−9.755	−0.635	−0.891	−1.188	−0.112
	Kurtosis	3.600	114.078	2.086	3.119	5.135	1.769
	Jarque-Bera	3.505	0.00096	18.54	22.9	73.28	11.58
	Probability	0.1734	0.000a	0.000a	0.000a	0.000a	0.000a
	lnHCE	1.000					
	lnGDP	0.167	1.000				
		(0.026)b					
	lnAGP	−0.107	0.109	1.000			
		(0.153)	(0.147)				
	lnURB	0.397	−0.102	−0.648	1.000		
		(0.000)a	(0.173)	(0.000)a			
	lnMT	0.322	0.021	0.012	0.165	1.000	
		(0.000)a	(0.782)	(0.879)	(0.027)		
	lnIND	0.037	0.221	0.748	−0.525	0.321	1.000
		(0.619)	(0.003)a	(0.000)a	(0.000)a	(0.000)a	
LMI	Mean	3.997	3.570	12.746	3.855	4.088	19.246
	Std. dev	0.608	0.099	1.499	0.141	0.115	6.526
	Variance	0.370	0.010	2.248	0.020	0.013	42.585
	Min	2.765	3.281	9.974	3.551	3.834	3.765
	Max	5.646	3.899	15.523	4.193	4.290	25.626
	Skewness	0.114	0.098	−0.114	0.487	−0.234	−1.756
	Kurtosis	2.583	4.234	2.642	2.828	2.385	4.597
	Jarque-Bera	1.313	9.106	1.053	5.714	3.487	86.86
	Probability	0.5186	0.0105b	0.5907	0.0574c	0.1749	0.000a
	lnHCE	1.000					
	lnGDP	0.070	1.000				
		(0.414)					
	lnAGP	−0.195	0.114	1.000			
		(0.021)b	(0.181)				
	lnURB	0.734	0.015	−0.509	1.000		
		(0.000)a	(0.864)	(0.000)a			
	lnMT	0.424	0.036	−0.789	0.686	1.000	
		(0.000)a	(0.677)	(0.000)a	(0.000)a		
	lnIND	−0.500	0.039	0.719	−0.684	−0.626	1.000
		(0.000)a	(0.647)	(0.000)a	(0.000)a	(0.000)a	
Whole sample	Mean	3.669	3.554	12.372	3.679	4.047	19.708
	Std. dev	0.636	0.242	1.224	0.322	0.106	4.437
	Variance	0.405	0.058	1.499	0.104	0.011	19.691
	Min	2.018	−0.157	9.974	2.784	3.675	3.765
	Max	5.646	4.050	15.523	4.193	4.290	25.626
	Skewness	0.156	−11.705	0.195	−1.345	−0.241	−2.714
	Kurtosis	3.171	176.479	3.329	4.681	3.596	10.414
	Jarque-Bera	1.694	410,000	3.474	134.1	7.827	1126
	Probability	0.4287	0.000a	0.176	0.000a	0.020b	0.000a
	lnHCE	1.000					
	lnGDP	0.139	1.000				
		(0.013)b					
	lnAGP	−0.014	0.095	1.000			
		(0.796)	(0.090)c				
	lnURB	0.587	−0.049	−0.269	1.000		
		(0.000)a	(0.378)	(0.000)a			
	lnMT	0.471	0.039	−0.362	0.392	1.000	
		(0.000)a	(0.482)	(0.000)a	(0.000)a		
	lnIND	−0.344	0.052	0.716	−0.332	−0.421	1.000
		(0.000)a	(0.355)	(0.000)a	(0.000)a	(0.000)a	

*a, b, and c denote significance at the 1, 5, and 10% levels, respectively; LI means low-income economies; LMI means lower-middle-income economies*.

We further conducted a Jarque-Bera test to examine if the data sample's skewness and kurtosis were normally distributed in the three panels. According to the results in the LI sample, healthcare expenditure was normally distributed, supporting the null hypothesis. In contrast, economic growth, aged population, urbanization, medical technology, and industrialization were not normally distributed, disproving the null hypothesis. In the LMI panel, healthcare expenditure, aged population, and medical technology values were normally distributed, thus, supporting the null hypothesis. Meanwhile, economic growth, urbanization, and industrialization were not normally distributed, disproving the null hypothesis. In the whole sample panel, healthcare expenditure and aged population were normally distributed, supporting the null hypothesis, whereas economic growth, urbanization, and medical technology were not normally distributed, thereby refuting the null hypothesis.

Table 3 again denotes the correlation among the study variables. From the outcome in the LI countries, there was a positive and significant relationship between economic growth and healthcare expenditure at a 5% significant level (*r* = 0.167, *p* < 0.05). Nonetheless, there was an adverse and insignificant connection between the aged population and healthcare expenditure (*r* = −0.107, *p* = 0.153). Additionally, there was a positive and material relationship between urbanization and healthcare expenditure (*r* = 0.397, *p* < 0.01). Similarly, the connection between medical technology and healthcare expenditure was positive and substantial (*r* = 0.322, *p* < 0.01). Lastly, the connection between industrialization and healthcare expenditure was positive but statistically irrelevant (*r* = 0.037, *p* = 0.619).

Besides, in the LMI countries, economic growth and healthcare expenditure had insignificant effects (*r* = 0.07, *p* = 0.414). Additionally, there was a negative and significant relationship between the aged population and healthcare expenditure (*r* = −0.195, *p* < 0.05). Moreover, a positive link between urbanization and healthcare expenditure was affirmed at a significant level of 1% (*r* = 0.734, *p* < 0.01). Similarly, a positive link between medical technology and healthcare expenditure was confirmed at a significant level of 1% (*r* = 0.424, *p* < 0.01). On the contrary, there was a negative and significant association between industrialization and healthcare expenditure (*r* = −0.5, *p* < 0.01).

Finally, Table 3 also reveals the correlations among the study variables in the whole sample. From the revelation, economic growth had a positive and material affiliation with healthcare expenditure (*r* = 0.139, *p* < 0.05). The connection between the aged population and healthcare expenditure was adverse and statistically irrelevant (*r* = −0.014, *p* = 0.796). However, the connection between urbanization and healthcare expenditure was positive and substantial (*r* = 0.587, *p* < 0.01). Likewise, there was a positive and material connection between medical technology and healthcare expenditure (*r* = 0.471, *p* < 0.01). The link between industrialization and healthcare expenditure was adverse and statistically relevant (*r* = −0.344, *p* < 0.1).

A strongly correlated variable can lead to inaccurate regression results, which can lead to skewed judgments, as postulated by Ahakwa et al. (51), Odai et al. (52), Korankye et al. (53), and Ahakwa et al. (54). As a result of this discovery, we performed a multi-collinearity assessment to determine if the variables were strongly linked or not. Variance inflation factor (VIF) and tolerance (1/VIF) assessments were performed for this experiment. Table 4 affirms that the VIF assessment results are within the required threshold, significant at 10 or less in all three panels. Also, the 1/VIF assessment results revealed that all the series exceeded the threshold of 0.1 in all the three panels, supporting those of Musah et al. (34), Gokmen et al. (55), and Zuur et al. (56). Also, the correlation matrix test to check for the collinearity problem was carried out, and as seen in Table 3, all correlation coefficients among the variables in all three panels are <0.8 (55). The tests mentioned above showed that the series could be used in this research assessment, collaborating with the results of Ahakwa et al. (57), Quagraine et al. (58), Ying et al. (59), Tackie et al. (60), and Ahakwa et al. (61).

**Table 4:** Multi-collinearity test results.

**Variables**	**LI**		**LMI**		**Whole Sample**	
	**VIF**	**1/VIF**	**VIF**	**1/VIF**	**VIF**	**1/VIF**
lnIND	5.963	0.168	5.798	0.172	2.229	0.449
lnAGP	5.611	0.178	7.874	0.127	2.088	0.479
lnMT	1.834	0.545	5.212	0.192	1.351	0.740
lnURB	1.653	0.605	3.577	0.280	1.236	0.809
lnGDP	1.105	0.905	1.093	0.915	1.019	0.982
Mean VIF	3.233	.	4.711		1.585	.

### Cross-Sectional Dependence and Slope Heterogeneity Test

Cross-country connections are characterized by a high degree of interdependence, owing to nations' increasing economic integration. Cross-sectional dependency is a significant issue in panel data analysis, and ignoring it may result in erroneous conclusions according to Musah et al. (62) and Guoyan et al. (63). Given this, the econometric analytical process began by determining whether or not the panel had cross-sectional reliability. The findings revealed dependencies in all the panels (see Table 5). As a result, we rejected the null assumption of cross-sectional independence. Based on the result, we employed econometric methods that are more robust in terms of cross-sectional reliance.

**Table 5:** Residual cross-sectional dependence test.

**Test method**	**LI**		**LMI**		**Whole Sample**	
	**Stat**	***p*-value**	**Stat**	***p*-value**	**Stat**	***p*-value**
Breusch-Pegan LM	3.114	0.0000a	109.74	0.0000a	151.890	0.0000a
Pesaran (36)	4.396	0.0000a	10.662	0.0000a	29.931	0.0000a
Friedman	38.797	0.0000a	71.049	0.0000a	170.907	0.0000a
Pesaran (37)	257.720	0.0000a	10.623	0.0000a	24.787	0.0000a

*a denotes significance at the 1% level.

The researchers examined whether the path coefficient was heterogeneous or uniform. Musah et al. (64) and Musah et al. (35) posited that unawareness of slope heterogeneity might bias regression outcomes and result in incorrect hypothesis testing. Consequently, the investigators employed the Pesaran and Yamagata (40) homogeneity test to address the issue mentioned above. The assessment results are shown in Table 6 and indicate that the null hypothesis of slope coefficient homogeneity is unsupported in all three panels. Based on these findings, the empirical analysis of the research adopted econometric methods that are resistant to slope heterogeneity.

**Table 6:** Pesaran-Yamagata homogeneity test results.

**Test**	**LI**		**LMI**		**Whole Sample**	
	**Value**	***p*-value**	**Value**	***p*-value**	**Value**	***p*-value**
Delta tide	6.345	0.000a	5.798	0.000a	−2.878	0.004a
Adjusted Delta tide	7.870	0.000a	7.191	0.000a	−3.838	0.000a

*a denotes significance at the 1% level.

### Unit Root Result

The stationarity of data could be determined by conducting a unit root evaluation. With the assumption that the study's panel data have a common unit process, investigations on the CIPS and CADF panel unit root assessments were performed, and the outcome is displayed in Table 7. Based on the outcome of the CIPS and CADF panel integration order assessments, it is possible to conclude that the null hypothesis of non-stationarity is unsupported for some variables at levels, and in the first difference form, it was not statistically significant for other series of variables. In conclusion, there was a mixed bag of outcomes in the order of integration of the variables between I(0) and I(1). This pattern in the outcomes is visible across the various panels, and this shows that the data series used in the analysis is insufficient to identify the unit root impact on the research variables. Based on the above, the unit root tests conducted could only serve as a roadmap to the test; still, they must not be totally depended upon as they can partially mitigate the issue of stationarity. The discrepancies in the integration order of the studied variables, at level forms and first difference, highlighted the fact that the correlation of the co-integration could not be formally detected. Consequently, the long-run effect interaction was estimated by the PMG-ARDL assessment in the subsequent section. The use of the approach mentioned above was backed by Pesaran et al. (49), who stated that ARDL is suitable in a case where the variables' integration order is single, thus I(0), I(1), or mixed.

**Table 7:** CIPS and CADF unit test results.

**Panel**		**CIPS**				**CADF**			
	**Variables**	**Level**	**Decision**	**First difference**	**Decision**	**Level**	**Decision**	**First difference**	**Decision**
LI	lnHCE	−1.788	I(0)	−3.616	I(1)	−1.823	I(0)	−2.635	I(1)
	lnGDP	0.098	I(0)	−0.892	I(0)	−2.239	I(1)	−2.895	I(1)
	lnIND	−1.590	I(0)	−3.990	I(1)	−1.663	I(0)	−3.028	I(1)
	lnMT	−1.230	I(0)	−2.294	I(1)	−5.973	I(1)	−4.938	I(1)
	lnURB	0.009	I(0)	−0.672	I(0)	−1.001	I(0)	−1.166	I(0)
	lnAGP	−4.371	I(1)	−5.780	I(1)	−2.712	I(1)	−2.112	I(0)
LMI	lnHCE	−2.185	I(0)	−4.499	I(1)	−1.877	I(0)	−3.197	I(1)
	lnGDP	−2.919	I(1)	−5.203	I(1)	−1.682	I(0)	-−2.430	I(1)
	lnIND	−1.527	I(0)	−3.349	I(1)	−1.495	I(0)	−2.466	I(1)
	lnMT	−2.565	I(0)	−1.306	I(0)	−6.063	I(1)	−5.151	I(1)
	lnURB	−1.550	I(0)	−2.818	I(1)	−0.495	I(0)	−2.797	I(1)
	lnAGP	−1.199	I(0)	−2.187	I(0)	−3.177	I(1)	−1.291	I(0)
Whole sample	lnHCE	−2.382	I(0)	−4.189	I(1)	−2.199	I(0)	−2.757	I(1)
	lnGDP	−3.851	I(1)	−5.273	I(1)	−3.112	I(1)	−3.798	I(1)
	lnIND	−4.204	I(1)	−5.862	I(1)	−1.872	I(0)	−3.008	I(1)
	lnMT	−2.489	I(0)	−2.920	I(1)	−5.222	I(1)	−4.341	I(1)
	lnURB	−1.659	I(0)	−2.267	I(0)	−2.771	I(1)	−2.640	I(1)
	lnAGP	−1.718	I(0)	−0.842	I(0)	−2.833	I(1)	−2.210	I(0)

### Model Estimation and Causality

The PMG-ARDL estimator calculated the long-term balance relationships in the series as presented in Table 8. Table 9 summarizes the manifestations and significance of the PMG-ARDL estimator. The findings showed that economic growth had a negative but insignificant effect on healthcare expenditure for all three panels. In addition, the aged population had a material and positive effect on healthcare expenditure in all three panels. The implication is that a rise in a unit of aged population increases healthcare expenditure by 2.975, 5.028, and 2.095%, respectively, in the whole sample and LMI and LI panels. Furthermore, it was discovered that urbanization had a positive but immaterial effect on healthcare expenditure in the LMI and LI panels. On the other hand, urbanization had a positive and significant influence on healthcare expenditure in the whole panel. Thus, a unit increase in urbanization will increase healthcare expenditure in West Africa by 2.614%. The study also revealed a positive and statistically insignificant relationship between healthcare expenditure and medical technology in the LI panel. Conversely, medical technology had a negative and substantive influence on healthcare expenditure in both the LMI panel and the whole sample. Finally, industrialization had a material and positive influence on healthcare expenditure in all three panels. This result implies that industrialization increases healthcare expenditure by 0.634, 0.729, and 0.348%, respectively, in the whole sample and LMI and LI panels.

**Table 8:** PMG-ARDL long-run estimation outcomes.

**Panel**	**Variables**	**Coefficient**	***t*-stat**	**Prob**
LI	lnGDP	−0.100	0.940	0.347
	lnIND	0.348	6.320	0.000a
	lnMT	2.365	1.540	0.125
	lnURB	0.919	1.360	0.175
	lnAGP	2.095	2.460	0.014b
LMI	lnGDP	−0.225	−1.170	0.241
	lnAGP	5.028	3.470	0.001a
	lnURB	1.582	0.930	0.355
	lnMT	−14.124	−4.260	0.000a
	lnIND	0.729	9.800	0.000a
Whole sample	lnGDP	−0.098	−0.630	0.527
	lnIND	0.634	12.060	0.000a
	lnMT	−8.797	−4.870	0.000a
	lnURB	2.614	4.450	0.000a
	lnAGP	2.975	4.140	0.000a

*a and b denote significance at the 1 and 5% levels, respectively; LI means low-income economies; LMI means lower-middle-income economies*.

**Table 9:** Summary of PMG-ARDL estimation results.

**Variable**	**LI**		**LMI**		**Whole Sample**	
	**Sign**	**Significance**	**Sign**	**Significance**	**Sign**	**Significance**
lnGDP	-	×	-	×	-	×
lnIND	+	√	+	√	+	√
lnMT	+	×	-	√	-	√
lnURB	+	×	+	×	+	√
lnAGP	+	√	+	√	+	√

**Figure 1:**
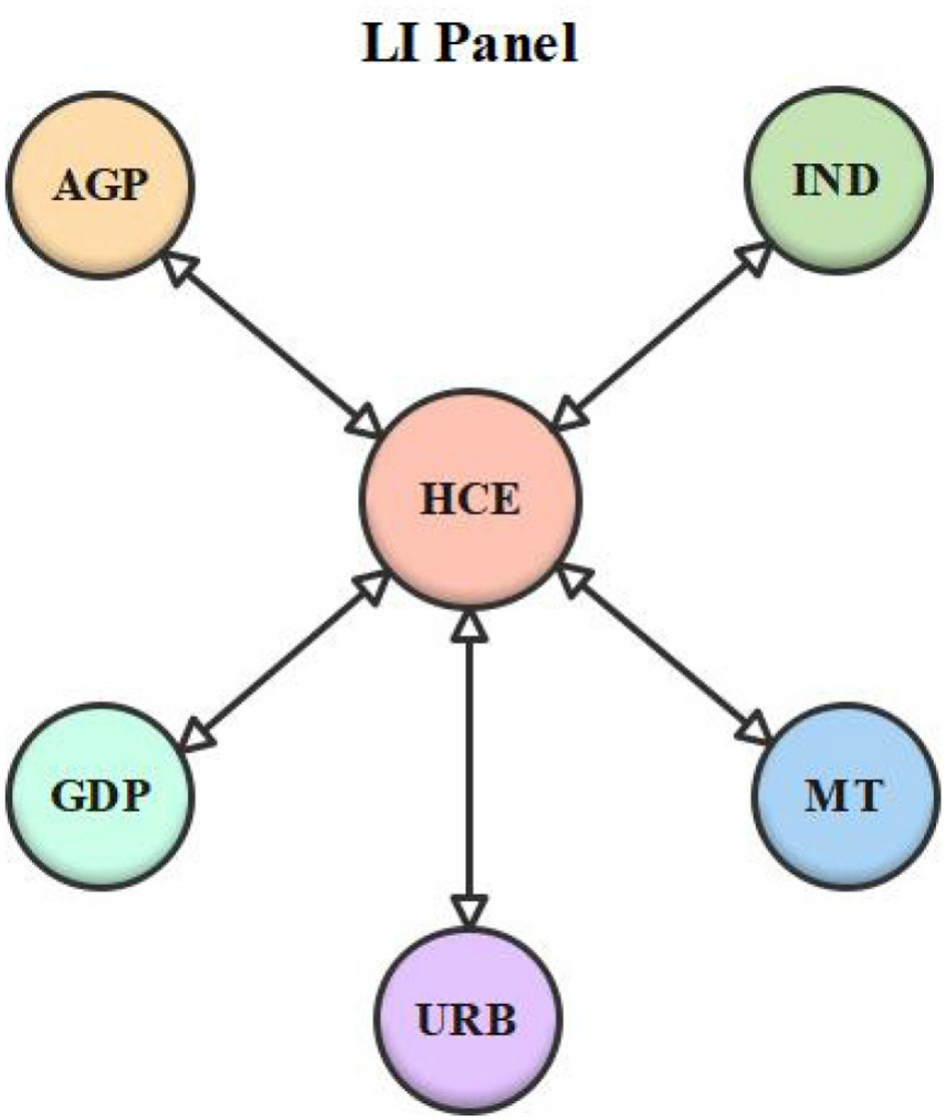
Low-income (LI) panel causality diagram.

**Figure 2:**
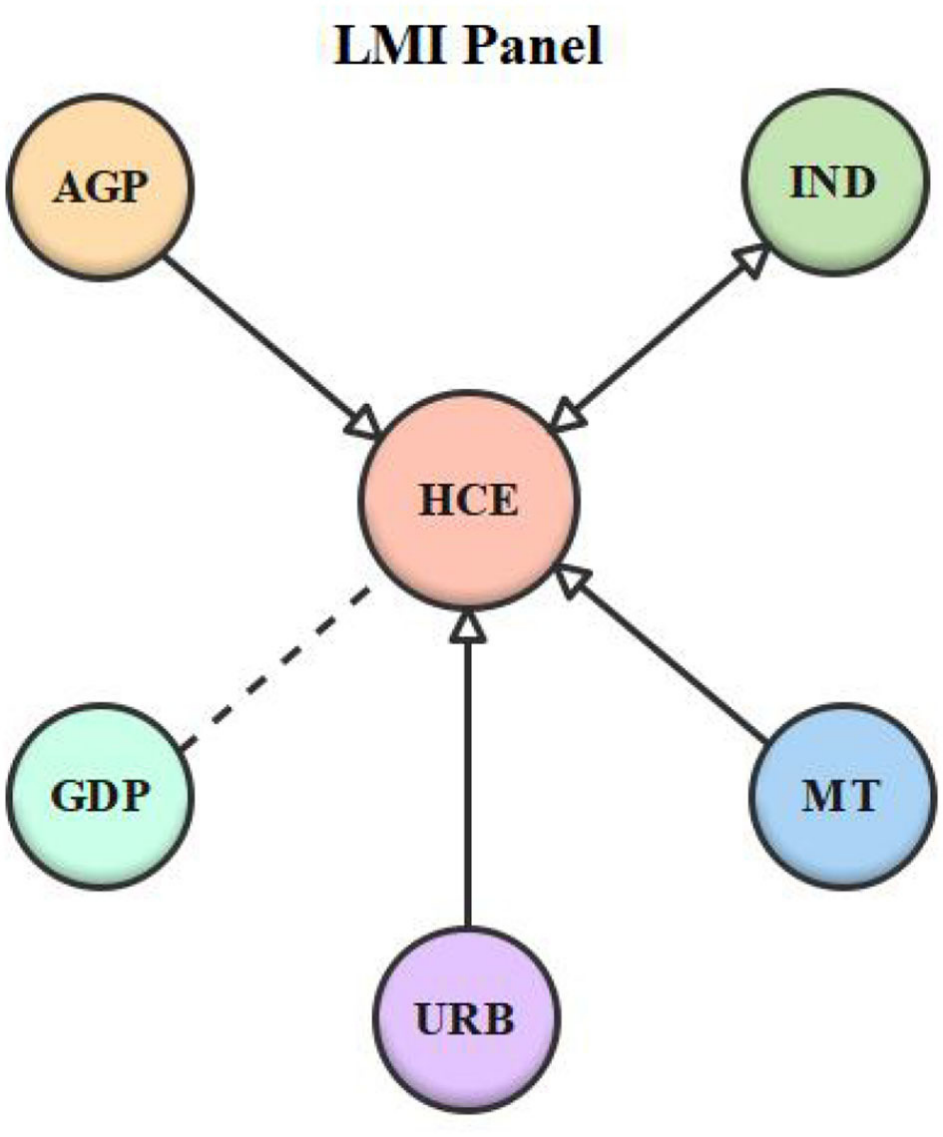
Lower-middle-income (LMI) panel causality diagram.

**Figure 3:**
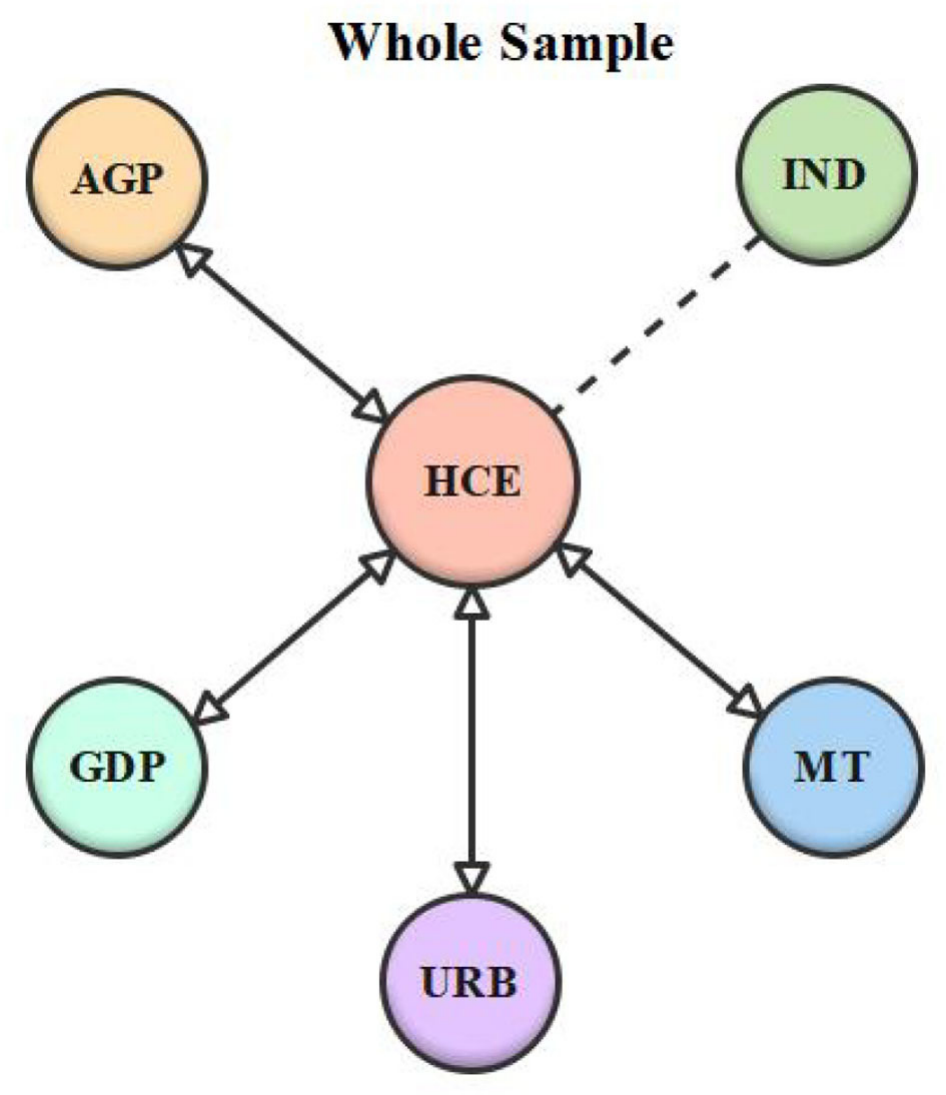
Whole sample causality diagram. “↔” represents feedback causality; “ → ” represents unidirectional causality, “…” represents no causality.

Lastly, a Dumitrescu and Hurlin (50) causality experiment was conducted to examine the causality among the series. Table 10 shows the test results, while Table 11 summarizes the causation conclusion. According to the findings, double-headed causality between economic growth and healthcare expenditure was found in the LI panel and the overall sample. In the LMI panel, however, there was no causal association between economic growth and healthcare expenditure. Besides, unidirectional causality between aged population and healthcare expenditure was detected in the LMI panel. Nonetheless, the LI panel and the entire sample confirmed a two-headed causal relationship between the aged population and healthcare expenditure. Moreover, there was double-headed causality between urbanization and healthcare expenditure in the LI panel and the whole panel. On the contrary, the results of the LMI panel revealed a unidirectional relationship between urbanization and healthcare expenditure, whereas, in the LMI panel, there was unidirectional causality between medical technology and healthcare expenditure. However, there was double-headed causality between medical technology and healthcare expenditure in both the overall sample and the LI panel. Finally, there was feedback causality between industrialization and healthcare expenditure in the LI and LMI panels, but no causal link between the two variables was affirmed in the overall sample. Figures 1–3 depict the series' causal relationships in the LI, LMI, and whole panels.

**Table 10:** Dumitrescu-Hurlin panel causality test results.

**Null hypotheses**	**LI**			**LMI**			**Whole Sample**		
	**W-Stat**	**zbar tilde**	**Prob**	**W-Stat**	**zbar tilde**	**Prob**	**W-Stat**	**zbar tilde**	**Prob**
lnGDP≪≫lnHCE	3.2411	3.4835	0.0005a	1.3466	0.2984	0.7654	2.4123	2.8100	0.0050a
lnHCE≪≫lnGDP	3.7862	4.3885	0.0000a	0.5841	−0.8182	0.4133	2.3853	2.7502	0.0060a
lnIND≪≫lnHCE	2.5946	2.4102	0.0159b	3.9961	4.1775	0.0000a	1.2100	0.0226	0.9820
lnHCE≪≫lnIND	3.3436	3.6537	0.0003a	3.2728	3.1185	0.0018a	0.6073	−1.3320	0.1829
lnMT≪≫lnHCE	19.3272	30.1895	0.0000a	29.0463	40.8550	0.0000a	23.5798	49.6664	0.0000a
lnHCE≪≫lnMT	2.6093	2.4346	0.0149b	1.8587	1.0482	0.2946	2.2810	2.5193	0.0118b
lnURB≪≫lnHCE	10.3516	15.2883	0.0000a	23.9921	33.4548	0.0000a	16.3094	33.5726	0.0000a
lnHCE≪≫lnURB	4.8796	6.2038	0.0000a	2.1108	1.4172	0.1564	3.6682	5.5900	0.0000a
lnAGP≪≫lnHCE	3.8541	2.8844	0.0038a	19.7717	27.2754	0.0000a	10.8305	21.4446	0.0000a
lnHCE≪≫lnAGP	3.8541	4.5012	0.0000a	2.2254	1.5850	0.1130	2.5961	3.2168	0.0013a

*a and b denote significance at the 1 and 5% levels, respectively; LI means low-income economies; LMI means lower-middle-income economies; ≪≫ denotes the null hypothesis that one variable does not homogeneously cause another variable*.

**Table 11:** Summary of Dumitrescu-Hurlin panel causalities.

**Hypotheses**	**LI**	**LMI**	**Whole sample**
	**Direction**	**Direction**	**Direction**
lnGDP≪≫lnHCE	↔	≠	↔
lnIND≪≫lnHCE	↔	↔	≠
lnMT≪≫lnHCE	↔	→	↔
lnURB≪≫lnHCE	↔	→	↔
lnAGP≪≫lnHCE	↔	→	↔

*“ → ” Indicates unidirectional causality from one variable to another, “↔” signposts bidirectional causality between variables, and “≠” denotes no causality between variables*.

## Discussion of Results

The PMG-ARDL estimator was used to assess the long-run effect connection among the variables. From the estimate, a negative but statistically immaterial nexus between economic growth and healthcare expenditure was affirmed in all three panels. One justification for the negative nexus is that as per capita income rises, people become wealthier, reducing economic hardship and improving physical and psychological wellbeing while decreasing demand for medical care. Nevertheless, this outcome was statistically irrelevant. The outcome supports Chireshe and Ocran (16), who conducted research on sub-Saharan African countries and found that government healthcare spending and economic growth are negatively connected. Again, the research supports Jakovljevic et al. (17), who maintain that economic growth does not escalate healthcare spending in Emerging Markets Seven (EM7) countries. The findings contradict those of Raghupathi and Raghupathi (12), who found a positive and substantial interaction between economic growth and healthcare expenditure in the United States. The findings also contradict Nyamwange (65), who discovered a significant positive association between Kenya's economic growth and healthcare expenditure. The outcome disapproves of Zhou et al. (5), who revealed that economic growth induces healthcare expenditure in developing countries. The findings likewise contradict the findings of Bakare and Olubokun (66), who discovered a positive relationship between economic growth and healthcare expenditure in Nigeria.

Moreover, the outcomes revealed that industrialization had a positive and substantial impact on healthcare expenditure in all three panels. This result is not surprising given that large-scale industrial complexes are static contributors to various environmental pollutants such as fine dust, sulfur dioxide, nitrogen dioxide, carbon monoxide, ozone, volatile organic compounds, heavy metals, and polycyclic aromatic hydrocarbons. Pollutants enter human cells *via* the cardiovascular system or the skin and cause allergies and other health conditions that negatively affect the human system (20). Healthcare expenditure is increased because of the health consequences of industrial activities. The findings back up Zhou et al. (5), who discovered a significant positive relationship between industrialization and healthcare expenditure in emerging economies. The findings further support Sagarik (67), who found a substantial positive connection between industrialization and healthcare expenditure in Southeast Asia.

Subsequently, medical technology had an adverse and material effect on healthcare expenditure in both the LMI panel and the entire sample according to the PMG-ARDL estimates. The negative effect of medical technology on healthcare expenditure means that an upsurge or a down-surge in medical technology would result in a major decrease or increase in healthcare spending and vice versa in the whole sample and LMI panel. The findings of this study indicate that medical technology plays an important role in national healthcare expenditure, as some diagnostic tests and drugs have become significantly less expensive, resulting in lower healthcare costs. On the other hand, medical technology had a positive but insubstantial effect on healthcare expenditure in the LI panel. LI countries have lower life expectancy than LMI countries. As a result, improving life expectancy, in the long run, necessitates the use of costly medical technologies and treatments. Healthcare expenditure is ineffective because of the lack of advanced medical technology in LI countries. Our LMI and overall sample findings contradict Jaba et al. (68), who found a strong positive relationship between healthcare expenditure and medical technology in a cross-country analysis. Our findings in the LI panel disapprove those of Duba et al. (69), who discovered a strong connection between medical technology and healthcare expenditure. The results, again, refute the findings of Shakoor et al. (22), who discovered a significant association between healthcare expenditure and medical technology in Pakistan.

Additionally, the results revealed that the aged population had a substantial and positive effect on healthcare expenditure in all the three panels. An upsurge or down surge in the aged population causes an increase or a reduction in healthcare expenditure, and vice versa, with a significant impact. This result is not surprising given that people's mental and physical health deteriorates as they grow, thus escalating healthcare expenditures. The aged population is also more susceptible to lasting diseases that require expensive treatments, which intensify the country's healthcare expenditure. This finding supported Lopreite and Mauro (70), who predicted that the elderly population's medical expenses are more costly than those of other age brackets. The findings support those of Nordin et al. (71), who discovered a long-term positive influence of the aged population on healthcare expenditure in developing countries. Likewise, a study conducted by Zhou et al. (5) confirms this study, revealing that the aged population causes healthcare expenditure in developing countries. Also, Lopreite and Zhu (27) discovered that the aged population causes healthcare expenditure to increase in China, supporting this study's findings. The findings contradict Baharin and Saad (30), who discovered that the aged population did not predict healthcare expenditure in Thailand.

Again, in the LI and LMI panels, urbanization was discovered to have a positive but minor effect on healthcare expenditure. The results suggest that an increase or a decrease in urbanization has no consequence on healthcare expenditure and vice versa in the LI and LMI panels. Urbanization, on the contrary, was found to have a positive and substantial effect on healthcare expenditure in the entire sample. This outcome in the whole sample can be connected to the fact that fast-expanding populations in big metropolises can intensify the transmission of infectious ailments, and that inadequate sanitary amenities could be unable to avert the potential healthcare cost of this transmission. Besides, pollution levels may rise in the quest to meet the increasing energy demands of people living in metropolises. As a result, it is not surprising to anticipate that healthcare spending in West Africa will rise. The findings are consistent with those of Çetin and Bakirtaş (24), who discovered urbanization as a significant predictor of healthcare expenditure in developing nations. The results support the findings of Kouassi et al. (72), who revealed that increased urbanization has a significant effect on healthcare expenditure in 14 Southern African Development Community (SADC) nations. The findings affirm that of Boachie et al. (73), who discovered that high rate of urbanization harmfully affects Ghana's public healthcare expenditures. On the other hand, the findings disprove Abbas and Hiemenz (74), who found that high levels of urbanization decrease government healthcare expenditure in Pakistan.

The PMG-ARDL estimator can only evaluate long-run equilibrium relationships among variables, because it cannot investigate causal relationships between variables. In light of this restraint, the Dumitrescu and Hurlin (50) causality assessment was conducted to determine the causal links among the explored series. From the outcome, according to the LMI panel, there was no causal connection between healthcare expenditure and economic growth. However, the entire sample and the LI panel revealed two-headed causality between economic growth and healthcare expenditure. This suggests that, in both the LI panel and the whole sample, healthcare expenditure was promoted by increases in the countries' economic growth, and the opposite holds, too. Likewise, any effort to reduce healthcare expenditure may lower the speed of economic progress of countries. This observation is comparable to Bedir (75), who discovered two-way causality between healthcare expenditure and economic growth in the Czech Republic and Russian Federation. The findings are in contrast to those of Ye and Zhang (76), who discovered causation that is unidirectional, flowing between economic growth and healthcare expenditure in Ireland, Korea, Portugal, and India. The findings further contradict Alhowaish (77), who demonstrated in Saudi Arabia a one-way causal relationship between economic growth to healthcare expenditure.

In addition, feedback causality between the aged population and healthcare expenditure in the LI panel and the entire sample was revealed. This suggests that the aged population and healthcare expenditure policies should be aligned. Also, in the LMI countries, unidirectional causality between the aged population and healthcare expenditure was proven. This could be influenced by the fact that the aged population's general health could deteriorate, resulting in chronic conditions or impairments, evidence that the aged population is a substantial reason for increased healthcare expenses. The results contradict the findings of Zhou et al. (5), who discovered unidirectional causality between healthcare expenditure and the aged population in growing economies. The findings contradict those of Ikeda et al. (78), who found that increase in the aged population does not always result in healthcare expenditure. Also, two-way causation between urbanization and healthcare expenditure was discovered in both the LI panel and the total sample. This finding suggests that healthcare expenditure and urbanization were so inextricably linked that a rise or a decline in one factor could lead to a rise or a drop in the other. Furthermore, the LMI panel confirmed a one-way causal link between urbanization and healthcare expenditure. This means that the migration of people to cities, which leads to increased industrialization, new investment, and infrastructure development, intensifies the countries' healthcare expenditure.

Moreover, the LI panel and overall sample revealed feedback causation between healthcare expenditure and medical technology. This suggests that healthcare expenditure and medical technology were surrounded by a consensual connection, such that a change in one variable caused a change in the other. In addition, a linear connection between medical technology and healthcare expenditure was established in the LMI panel. The LI panel and whole sample findings are consistent with Ogunsakin and Olonisakin (79), who discovered two-way causality between healthcare expenditure and medical technology in Nigeria. The research results also back the findings of Zhou et al. (5), who found two-headed causality between healthcare expenditure and medical technology in growing economies. The findings contend with Nkemgha et al. (80), who discovered unidirectional causality between medical technology and healthcare expenditure in Cameroon. The findings again contradict Ogungbenle et al. (81), who discovered no causality between medical technology and healthcare expenditure in Nigeria.

Finally, the LI and LMI panels revealed two-way causation between industrialization and healthcare expenditure. This finding suggests that healthcare expenditure and industrialization are so inseparably connected that a variation in one variable could result in an increase or a decline in the other. Subsequently, no causal link between industrialization and healthcare expenditure was established in the entire sample. The result contradicts Zhou et al. (5), who found single causality between healthcare expenditure and industrialization in growing economies.

## Conclusion and Policy Implications

The dynamic nexus among economic growth, industrialization, medical technology, and healthcare expenditure was investigated in this study while using urbanization and the aged population as control variables. The study, therefore, employed second-generation econometric methods robust to heterogeneity and cross-sectional units. According to the cross-sectional dependence and homogeneity test results, the panel sample used for this study was diverse and cross-sectional-dependent. Again, according to the unit root outcome, there was a mixed integration order at levels and after the first difference in all the three panels. Differences in the integration order of the research variables, both at the level forms and at the first difference, underline the fact that a co-integration correlation among the variables cannot be formally established. As a result of this problem, the study used the PMG-ARDL estimation technique to examine the long-run effect connection among the series. The findings revealed that economic growth is not a predictor of healthcare expenditure in West Africa. In addition, the aged population exacerbated the healthcare expenditure in West Africa. Furthermore, while urbanization increased healthcare expenditure in the entire sample, it was insignificant in the LI and LMI panels. Also, medical technology had a substantially negative effect on healthcare expenditure in the entire sample and the LMI panel, but it was statistically insignificant in the LI panel. Moreover, industrialization increased healthcare expenditure in all three panels. Lastly, the Dumitrescu-Hurlin causality test revealed bidirectional causality between economic growth and healthcare expenditure, aged population and healthcare expenditure, urbanization and healthcare expenditure, and medical technology and healthcare expenditure in the entire sample and LI panel. Also, the LMI panel created linear causality between aged population and healthcare expenditure, urbanization and healthcare expenditure, and medical technology and healthcare expenditure. Lastly, in the LI and LMI panels, feedback causation between industrialization and healthcare expenditure was confirmed. The study made the following recommendation based on its findings:

1. First and foremost, West African policymakers should revisit their urbanization strategies in order to prevent the possible harmful consequences of a rapid urban population increase. In addition, authorities must work to create jobs and improve the living conditions of rural residents. As a result, people will move from rural to urban areas at a slower rate. Furthermore, providing social services to rural locations will help reduce urbanization and healthcare expenditure.
2. According to the research, a rise in the older population contributes significantly to rising healthcare expenses in West Africa. Consequently, the study recommends that health officials develop, modify, and intensify health education programs focused on increasing health and illness prevention among the elderly. The aged population must not be regarded as a drain on the economy, because they are the most vulnerable; still, a healthy elderly population may add to economic progress by sharing and expanding their ideas and knowledge with today's youth.
3. Industrialization was found to escalate healthcare expenditure. Therefore, policymakers must implement policies that encourage environmental sustainability and economic growth in their various nations. This goal can be achieved by changing energy policy to reduce manufacturing activity's reliance on non-renewable energy sources while encouraging renewable energy sources. Renewable energy sources will reduce harmful emissions from industrial operations, help countries thrive economically, and lower healthcare expenditure.
4. Governments should support technological developments in the health sector in order to raise the standard of living in West Africa. As a result, policies aimed at providing quality healthcare by investing in advanced technologies such as the use of sophisticated equipment such as diagnostic items and quality drugs for treatments should be implemented. Investments in the health sector should be encouraged because they lead to increased human capital development and population welfare, which lead to economic growth. The current COVID-19 pandemic demonstrates the significance of investing in the health sector.

### Limitations

This study had two major flaws that needed to be addressed. To begin with, the researchers aimed to use a much longer time than what was used, but because of data restraints, the study used data from 2000 to 2019. When such data are fully available, the researchers encourage subsequent studies to report longer periods than the study term. Further research could look into other predictors of healthcare expenditure in West Africa such as agricultural activities, foreign direct investment, and carbon emission.

## Data Availability Statement

Publicly available datasets were analyzed in this study. This data can be found at: https://databank.worldbank.org/source/world-development-indicators.

## Author Contributions

ET conceptualized and wrote original and final manuscript. HC supervised the study, aided in discussions, and editing the final manuscript. IA aided in drafting the original manuscript and helped in analysis and discussions. SA aided in discussions and editing the final manuscript. All authors contributed to the article and approved the submitted version.

## Funding

This work was supported by the National Social Science Foundation of China (NSSFC; 18BGL255).

## Conflict of Interest

The authors declare that the research was conducted in the absence of any commercial or financial relationships that could be construed as a potential conflict of interest.

## Publisher's Note

All claims expressed in this article are solely those of the authors and do not necessarily represent those of their affiliated organizations, or those of the publisher, the editors and the reviewers. Any product that may be evaluated in this article, or claim that may be made by its manufacturer, is not guaranteed or endorsed by the publisher.
